# Dihydroartemisinin–piperaquine holds promise as an option for malaria prevention in pregnancy

**DOI:** 10.1136/ebmed-2016-110438

**Published:** 2016-05-20

**Authors:** Jenny Hill, Feiko O ter Kuile

**Affiliations:** Liverpool School of Tropical Medicine, Liverpool, UK

**Commentary on**: KakuruA, JagannathanP, MuhindoMK, et al Dihydroartemisinin-piperaquine for the prevention of malaria in pregnancy. N Engl J Med 2016;374:928–39.2696272810.1056/NEJMoa1509150PMC4847718

## Context

Malaria in pregnancy has devastating consequences for mother and fetus. WHO recommends intermittent preventive treatment in pregnancy (IPTp) with treatment doses of an efficacious antimalarial during the second and third trimesters of pregnancy at predefined intervals. Sulfadoxine–pyrimethamine is currently recommended, but high-level parasite resistance threatens its efficacy. Recent trials showed that amodiaquine, mefloquine and chloroquine–azithromycin are not suitable alternatives due to poor tolerability. This trial by Kakuru *et al* evaluated the artemisinin-based combination therapy (ACT), dihydroartemisinin–piperaquine, for IPTp. Dihydroartemisinin–piperaquine is already recommended by WHO for treatment of malaria in the second and third trimesters. A recent comparison of four ACTs for treatment of malaria in pregnancy in Africa showed that dihydroartemisinin–piperaquine had the best efficacy and a long post-treatment prophylactic effect, which supports its suitability for IPTp in high transmission areas.^1^

## Methods

The trial by Kakuru *et al* was a double-blind, randomised, controlled, three-arm trial comparing three-course dihydroartemisinin–piperaquine and monthly dihydroartemisinin–piperaquine versus the standard three-course sulfadoxine–pyrimethamine regimen as IPTp among HIV-uninfected pregnant women between 12 and 20 weeks gestation, using a 1:1:1 allocation. The study was conducted in an area of high sulfadoxine–pyrimethamine resistance in Uganda. The primary outcome was the prevalence of histopathology-confirmed placental malaria. Secondary outcomes included symptomatic malaria, parasitaemia, anaemia and adverse birth outcomes. Women and newborns were followed up to 6 weeks post delivery.

## Findings

Three hundred women were enrolled. The dihydroartemisinin–piperaquine regimens were well tolerated. Compared with sulfadoxine–pyrimethamine, three-course and monthly dihydroartemisinin–piperaquine regimens were associated with a 67% and 100% reduction in incidence of clinical malaria during pregnancy, respectively; a 32% and 46% reduction in malaria infection at delivery; and a 13% and 34% lower risk of anaemia at delivery. Compared with sulfadoxine-pyrimethamine, the relative risk of a composite adverse birth outcome was 0.49 (95% CI 0.23 to 1.04) in the monthly dihydroartemisinin–piperaquine arm, and slightly higher (RR 1.15 95% CI 0.65 to 2.02) in the three-course arm, compared with sulfadoxine–pyrimethamine. There were no differences in safety or adverse events by study arm.

## Commentary

This exploratory trial showed that IPTp with dihydroartemisinin–piperaquine was superior to sulfadoxine–pyrimethamine in reducing malaria infections during pregnancy in an area of high sulfadoxine–pyrimethamine resistance, however the study was not powered to detect differences in adverse birth outcomes. The results are consistent with another trial in western Kenya, which showed that, relative to IPTp with sulfadoxine–pyrimethamine, three-to-four courses of dihydroartemisinin–piperaquine resulted in similarly high reductions in clinical malaria and malaria infection, and was also associated with up to a 75% lower risk of stillbirths and early infant mortality.^2^ Interestingly, the Uganda trial also showed that monthly dosing results in added benefits over three-course dihydroartemisinin–piperaquine or sulfadoxine–pyrimethamine regimens, consistent with a meta-analysis of monthly versus two-course sulfadoxine–pyrimethamine.^3^

## Implications for practice

The WHO's Malaria Policy Advisory Committee reviewed the results from both Ugandan and Kenyan trials, and concluded that dihydroartemisinin–piperaquine is a promising regimen for IPTp, and recommended that larger trials be conducted, powered to look at efficacy on adverse pregnancy outcomes and to provide further reassurance on safety.^4^

Furthermore, questions remain regarding pregnant women's adherence to dihydroartemisinin–piperaquine, a 3-day regimen, in non-controlled settings.^5^ Similarly, feasibility studies on the ability of antenatal care service providers to deliver the intervention in routine healthcare settings are also needed, given that coverage with the existing single-dose policy with sulfadoxine–pyrimethamine is already suboptimal.^6^ It is estimated these studies may take 3–4 years. If these promising findings are confirmed, it is likely to result in policy change in countries experiencing high levels of parasite resistance, including most countries in East and Southern Africa, benefiting women at risk of malaria in these regions, and resulting in healthier pregnancies and healthier newborns.

## References

[1] Pekyi D, Ampromfi AA, Tinto H, et al, Pregact Study Group. Four artemisinin-based treatments in African pregnant women with malaria. N Engl J Med 2016;374:913–27. 10.1056/NEJMoa1508606 26962727

[2] Desai M, Gutman J, L'lanziva A, et al Intermittent screening and treatment or intermittent preventive treatment with dihydroartemisinin-piperaquine versus intermittent preventive treatment with sulfadoxine-pyrimethamine for the control of malaria during pregnancy in western Kenya: an open-label, three-group, randomised controlled superiority trial. Lancet 2015;386:2507–19. 10.1016/S0140-6736(15)00310-4 26429700 PMC4718402

[3] Kayentao K, Garner P, van Eijk AM, et al Intermittent preventive therapy for malaria during pregnancy using 2 vs 3 or more doses of sulfadoxine-pyrimethamine and risk of low birth weight in Africa: systematic review and meta-analysis. JAMA 2013;309:594–604. 10.1001/jama.2012.216231 23403684 PMC4669677

[4] WHO Malaria Policy Advisory Committee and Secretariat. Malaria Policy Advisory Committee to the WHO: conclusions and recommendations of eighth biannual meeting (September 2015). Malar J 2016;15:117 10.1186/s12936-016-1169-x 26911803 PMC4766637

[5] Hill J, Hoyt J, Achieng F, et al User and provider acceptability of intermittent screening and treatment and intermittent preventive treatment with Dihydroartemisinin-Piperaquine to prevent malaria in pregnancy in Western Kenya. PLoS ONE 2016;11:e0150259 10.1371/journal.pone.0150259 26986471 PMC4795545

[6] van Eijk AM, Hill J, Larsen DA, et al Coverage of intermittent preventive treatment and insecticide-treated nets for the control of malaria during pregnancy in sub-Saharan Africa: a synthesis and meta-analysis of national survey data, 2009–11. Lancet Infect Dis 2013;13:1029–42. 10.1016/S1473-3099(13)70199-3 24054085

